# Analgesic Efficacies of Intraoperative Pectoralis Nerve II Block under Direct Vision in Patients Undergoing Robotic Nipple-Sparing Mastectomy with Immediate Breast Reconstruction: A Prospective, Randomized Controlled Study

**DOI:** 10.3390/jpm12081309

**Published:** 2022-08-12

**Authors:** Jiae Moon, Hyung Seok Park, Jee Ye Kim, Hye Sun Lee, Soyoung Jeon, Dongwoo Lee, Sun Joon Bai, Na Young Kim

**Affiliations:** 1 Department of Anesthesiology and Pain Medicine, Anesthesia and Pain Research Institute, Yonsei University College of Medicine, Seoul 03722, Korea; 2 Department of Surgery, Yonsei University College of Medicine, Seoul 03722, Korea; 3 Department of Research Affairs, Biostatistics Collaboration Unit, Yonsei University College of Medicine, Seoul 03722, Korea

**Keywords:** pectoralis nerve II block, robotic nipple-sparing mastectomy, immediate breast reconstruction, opioid consumption, direct-to-implant, tissue expander, analgesia

## Abstract

This prospective, randomized study aimed to evaluate the efficacy of an intraoperative pectoralis nerve II block (PECS II block) under direct vision in the reduction of fentanyl consumption during postoperative 24 h in patients undergoing robotic nipple-sparing mastectomy (RNSM) with immediate breast reconstruction (IBR) using direct-to-implant (DTI) or tissue expander (TE). Thirty patients scheduled for RNSM with IBR were randomly allocated to the PECS (*n* = 15) or control (*n* = 15) groups. The PECS II block was applied under direct vision after RNSM. The primary outcome was the cumulative dose of fentanyl consumption. The secondary outcomes were pain intensity using a numerical rating scale (NRS) at rest and acting during the postoperative 24 h. The cumulative dose of fentanyl at 24 h was significantly lower in the PECS group than in the control group (*p* = 0.011). Patients in the PECS group showed significantly lower NRS scores during the first postoperative 2 h compared to those in the control group in both resting and acting pain (*p* < 0.05). An intraoperative PECS II block under direct vision can reduce opioid consumption during the postoperative 24 h and provide effective analgesia in patients undergoing RNSM with IBR using DTI or TE.

## 1. Introduction

The pectoralis nerve II block (PECS II block) under ultrasound guidance, first described by Blanco et al. [1], has been increasingly used for analgesia in breast surgery and has been reported to be effective in reducing postoperative pain intensity and opioid consumption [2,3]. It is safe and relatively simple to perform as an interfascial plane block, and there is no sympathetic blockade [4]. The PECS II block consists of two interfascial injections of local anesthetic: one between the pectoralis minor and serratus anterior muscles and the other between the pectoralis major and pectoralis minor muscles at the third rib level [1]. Injection of a local anesthetic into these planes is expected to anesthetize the lateral pectoral nerve, medial pectoral nerve, anterior divisions of the thoracic intercostal nerves, and long thoracic nerves [1,5,6].

Robotic nipple-sparing mastectomy (RNSM) with immediate breast reconstruction (IBR) has been increasingly performed since it was first introduced by Toesca et al. [7]. The delicate and precise operation of the robotic surgical system allows RNSM with IBR to be performed through a 2.5–6 cm linear mid-axillary incision below the axillary fossa [8]. Through this incision, an intraoperative PECS II block under direct vision can be performed by the surgeon. Furthermore, although several studies regarding intraoperative PECS block under direct vision through an open surgical incision have been reported [9,10,11], there are limited studies regarding the analgesic efficacy of an intraoperative PECS II block under direct vision in patients undergoing RNSM with IBR. Thus, this study aimed to evaluate the efficacy of intraoperative PECS II block under direct vision in the reduction of fentanyl consumption during postoperative 24 h in patients undergoing RNSM with IBR using direct-to-implant (DTI) or tissue expander (TE).

## 2. Materials and Methods

### 2.1. Study Populations

This prospective, randomized controlled study was approved by the Institutional Review Board and Hospital Research Ethics Committee of Yonsei University Health System, Seoul, Korea, and registered in a clinical trial registry (http://www.clinicaltrials.gov, identifier: NCT04440995, accessed on 22 June 2020). We enrolled 30 participants aged ≥20 years with American Society of Anesthesiologists (ASA) physical status I to III who were scheduled for RNSM with IBR between July 2020 and October 2021 after obtaining informed written consent. Patients who met any of the following criteria were excluded from the study: (1) history of allergy to the study medications; (2) coagulopathy; (3) use of anticoagulation therapy; (4) patient refusal to use patient-controlled analgesia (PCA); (5) body mass index (BMI) > 35 kg/m^2^; (6) history of uncontrolled hypertension, diabetes, heart failure, hepatic failure, renal failure, and/or cerebrovascular disease; and (7) history of uncontrolled psychiatric disease.

### 2.2. Randomization and Intervention

The participants were randomly assigned to either the PECS group (*n* = 15) or the control group (*n* = 15) according to a computer-generated randomization sequence. In the control group, patients underwent RNSM with IBR without PECS II block. In the PECS group, PECS II block was conducted at the end of the RNSM, followed by IBR. All RNSM and PECS II blocks were performed by a single surgeon, H.S.P.

A blunted tip cannula (27 G, 30 mm, Mirror Cannula, Namumcompany Co., Ltd., Miami, FL, USA) was inserted between the fascia of the pectoralis major and pectoralis minor muscles at the level of the third rib, which was exposed through the RNSM incision, and 10 mL of 0.25% ropivacaine (Nacain Injection; Huons Co., Sungnam, Korea) was injected under direct vision. The aim of administering this local anesthetic was to reduce myofascial pain from the pectoralis muscles by anesthetizing the lateral and medial pectoral nerves that pass medially toward the pectoralis major and minor muscles [6]. The pectoralis minor and serratus anterior muscles were identified at the level of the third rib, and 20 mL of 0.25% ropivacaine was directly injected into the interfacial space using a blunted tip cannula. This injection targets the long thoracic nerve and the two or three lateral cutaneous branches of the thoracic intercostal nerve to provide postoperative analgesia by anesthetizing the axilla and lateral chest wall [6]. The 30 mL of 0.25% ropivacaine was prepared by a nurse who did not participate in this study.

### 2.3. Procedures

#### 2.3.1. Anesthesia

When the patient arrived in the operating room, the following monitoring devices were mounted on the patient: noninvasive blood pressure, electrocardiography, oxygen saturation, peripheral nerve stimulator, and patient state index using a SedLine^®^ electroencephalograph sensor (Masimo Corp., Irvine, CA, USA). Then, 0.1 mg of glycopyrrolate was administered intravenously (IV) as a premedication; thereafter, anesthesia was induced with propofol 1–2 mg/kg and remifentanil 0.05–0.1 µg/kg. Tracheal intubation was facilitated with rocuronium 0.6 mg/kg after loss of consciousness. Mechanical ventilation was started with an inspiratory–expiratory ratio of 1:2, positive end-expiratory pressure of 5 cm H2O, and target tidal volume of 8 mL/kg ideal body weight. The respiratory rate was adjusted to maintain the end-expiratory carbon dioxide between 35–42 mmHg. Anesthesia was maintained with remifentanil 0.03–0.1 µg/kg/min and sevoflurane (0.6–1.0 age-adjusted minimum alveolar concentration). Postoperative neuromuscular blockade was evaluated using a nerve stimulator and antagonized with IV sugammadex (Bridion^®^, MSD, Seoul, Korea).

#### 2.3.2. RNSM

The RNSM procedure has been described in detail in previous studies. In brief, a 2.5–6 cm incision was made longitudinally in the mid-axillary line below the axillary fossa. After the manual preparation of the working space, robotic system docking, robotic dissection, and specimen retrieval were performed [7,8,12,13,14]. After specimen retrieval, the PECS II block was performed using a blunted tip cannula via the incision by a single surgeon.

#### 2.3.3. IBR

After robotic mastectomy, IBR was performed by the plastic surgeons using TE or a smooth, round, stable gel mammary implant covered with an acellular dermal matrix. A covered implant or a TE was inserted in the prepectoral plane [12,15].

### 2.4. Postoperative Pain Management

During subcutaneous tissue suturing, 1 µg/kg of IV fentanyl (Hana Pharm, Seoul, Korea) for postoperative pain control and 0.3 mg of IV ramosetron (Nasea^®^, Astellas Pharma Korea, Seoul, Korea) for postoperative nausea and vomiting (PONV) prevention were administered. All patients in both groups received IV-PCA (Accumate 1200; WooYoung Medical, Seoul, Korea), which was a mixture of a total volume of 250 mL, consisting of 5 µg/kg of fentanyl, 0.3 mg of ramosetron, and 0.9% normal saline. All PCA devices were set to deliver a bolus dose of 10 mL (fentanyl: 0.2 µg/kg), with a 15-min lock out interval and a basal infusion rate of 0.1 mL (fentanyl: 0.002 µg/kg/h). Instructions on how to present the intensity of pain using a numerical rating scale (NRS; 0, no pain; 10, worst pain possible) and how to use the PCA device were provided to all enrolled patients in the preanesthetic room [16]. After the patients were transferred to the postanesthetic care unit and had emerged from anesthesia, the recovery nurses who were not involved in the study assessed pain intensity using NRS scores. The patients were reinstructed about the use of the PCA machine and were encouraged to press the bolus button whenever they felt pain greater than NRS score 3. When the patient experienced sustained pain (NRS score ≥ 4), 50 µg of IV fentanyl was administered as an additional rescue analgesic. After being transferred to the ward, all patients were assessed by the PCA management team of our institution, who was unaware of group assignments and had no involvement in anesthesia or surgical procedures. In the ward, all patients received 1 g of IV paracetamol (profa^®^, Dai Han Pharm, Seoul, Korea) and one tablet of Mypol^®^ (codeine phosphate 10 mg plus ibuprofen 200 mg, Sung-won Adcock Pharm, Seoul, Korea) every 8 h for 24 h. In patients who suffered from prolonged pain with an NRS score of ≥4 in the ward even after repeated administration of the PCA bolus, 50 mg of tridol (Tramadol HCL^®^, Yuhan. Co., Seoul, Korea) was administered as an additional analgesic. The investigator, recovery nurses, PCA management team, and patients were blinded to the group allocation.

### 2.5. Outcomes

The primary endpoint of this study was the cumulative dose of fentanyl consumed during the 24 h after surgery. The amount of fentanyl consumed was evaluated using data stored in the PCA device. The secondary endpoints were the resting and acting pain intensity using NRS at 0, 1, 2, 4, 6, 8, 12, and 24 h after surgery. Resting pain was defined as pain at rest or lying still, and acting pain was defined as pain when moving or changing posture or coughing [17].

### 2.6. Data Collection

Patient characteristics, including age, BMI, ASA physical status, smoking status, menopause status, history of motion sickness and PONV, and neoadjuvant chemotherapy were recorded. The following intraoperative and surgical characteristics were assessed: surgical location, type of reconstruction, type of lymph node procedure, specimen weight, duration of anesthesia and operation, mastectomy and reconstruction time, intraoperative blood loss and flood input, intraoperative urine output, and dose of administered remifentanil and phenylephrine.

Postoperative data included length of postoperative hospital stay, patient satisfaction score, PONV, and complications. Patient satisfaction was assessed on a 4-point scale reported by the patients (patient satisfaction score 1 = very unsatisfied, 4 = very satisfied) at postoperative 24 h [18,19]. Postoperative nausea was assessed using a 4-point scale (0–3; 0 = none, 1 = mild, 2 = moderate, and 3 = severe), and vomiting was recorded 24 h after surgery. Block-related or postoperative complications were recorded until discharge.

### 2.7. Statistical Analysis

In a preliminary study, the total amount of fentanyl administered via PCA for 24 h after RNSM with IBR was 252.3 ± 142.0 μg. To detect a 60% reduction in fentanyl consumption, a sample size of 15 patients in each group was required to have an 80% power, with an alpha error of 0.05, considering a potential dropout rate of 5%.

Continuous variables were expressed as mean ± standard deviation and analyzed using Student’s *t*-test. Categorical variables were expressed in terms of the number of patients (percentage), and the chi-square or Fisher’s exact test was performed. Repeatedly measured variables, such as cumulative fentanyl consumption and NRS score for resting and acting pain, were analyzed using a linear mixed model. Post hoc analysis was performed and adjusted using the Bonferroni method. Statistical significance was set at *p* < 0.05. All statistical analyses were performed using SAS version 9.4 (SAS Inc., Cary, NC, USA).

## 3. Results

**Figure 1:**
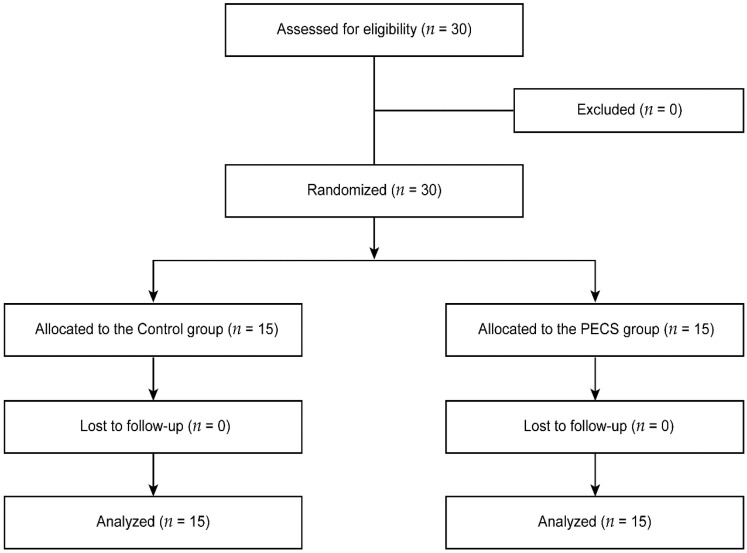
Consolidated Standards of Reporting Trials flow diagram. PECS, pectoralis nerve II block.

A total of 30 patients were evaluated for eligibility and were randomly assigned into two groups. All patients completed the study and were included in the final analysis (Figure 1).

Patient characteristics are shown in Table 1. The number of patients with a history of motion sickness was significantly higher in the PECS group than in the control group (*p* = 0.042). There were no differences in the other variables between the two groups.

The intraoperative and surgical characteristics of the enrolled patients in both the groups were comparable (Table 2).

**Figure 2:**
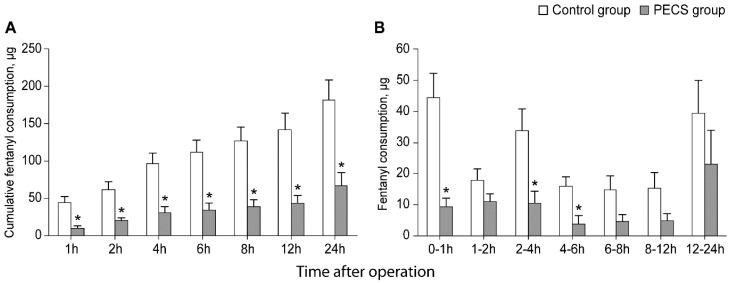
Dose of cumulative fentanyl consumption (**A**) and fentanyl consumption during 24 h after operation (**B**). Values are presented as mean ± standard error. PECS, pectoralis nerve II block. * Bonferroni corrected *p <* 0.05 versus the control group.

Figure 2 demonstrates the doses of fentanyl administered via IV-PCA during postoperative 24 h in both groups. Significant group differences were observed in cumulative fentanyl consumption at all-time points in the linear mixed model analysis (Bonferroni corrected *p* = 0.001, 0.006, 0.004, 0.002, 0.002, 0.003, and 0.011 at 1, 2, 4, 6, 8, 12, and 24 h, respectively) (Figure 2A). The cumulative fentanyl amounts at 24 h was 67 ± 23 µg in the PECS group and 182 ± 23 µg in the control group. Patients in the PECS group required significantly less fentanyl compared to those in the control group at postoperative 0–1 h, 2–4 h, and 4–6 h (Bonferroni corrected *p* = 0.001, 0.043, and 0.028, respectively), which was 80%, 71%, and 75% lower in the PECS group than in the control group (Figure 2B).

**Figure 3:**
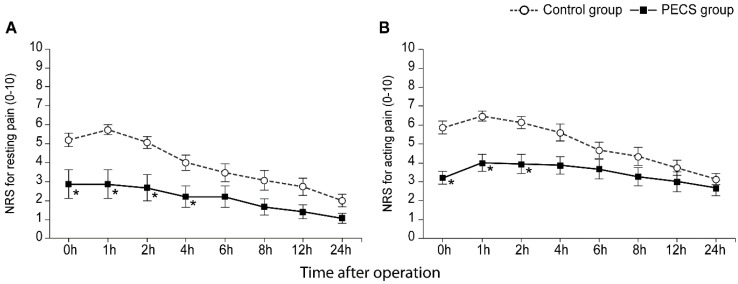
Numerical rating scale for resting (**A**) and acting (**B**) pain intensity during 24 h after operation. Values are presented as mean ± standard error. PECS, pectoralis nerve II block; NRS, numerical rating scale. * Bonferroni corrected *p <* 0.05 versus the control group.

The NRS scores for resting and acting pain intensities are shown in Figure 3A,B, respectively. Patients in the PECS group showed significantly lower NRS scores during the first postoperative 2 h compared to those in the control group in both resting and acting pain (all Bonferroni corrected *p* < 0.05). However, in resting pain intensity, the NRS score was significantly lower in the PECS group up to 4 h after surgery (Bonferroni corrected *p* = 0.024) (Figure 3A).

The patient satisfaction score on postoperative day 1 was significantly higher in the PECS group than in the control group (*p* = 0.002). No intergroup differences were observed in the incidence of PONV (Table 3). In addition, no patients who suffered from any block-related or postoperative complication were observed.

## 4. Discussion

This prospective, randomized, controlled trial was the first to evaluate the analgesic efficacy of the intraoperative PECS II block under direct vision in patients undergoing RNSM with prepectoral IBR using DTI or TE. Our findings suggest that intraoperative PECS II block under vision reduced cumulative fentanyl consumption by 63% during postoperative 24 h. In addition, the PECS II block provided better analgesia based on significantly lower NRS scores in both resting and acting pain during the first two postoperative hours.

Various reports have shown that a preoperative ultrasound-guided PECS II block reduces total opioid consumption in the first 24 h after major breast cancer surgery [4,20,21,22,23,24]. Consistent with these findings, we found that the cumulative dose of fentanyl required during postoperative 24 h was significantly lower in the PECS group receiving an intraoperative PECS II block under vision than in the control group (67 ± 23 µg versus 182 ± 23 µg, respectively), especially in the postoperative 0–1 h, 2–4 h, and 4–6 h. However, fentanyl consumption was not significantly different between the two groups after postoperative 6 h. Versyck et al. [2] reported in a meta-analysis study that the use of the PECS II block reduces postoperative pain scores at all-time points assessed up to 24 h. Similarly, a meta-study by Lovett-Carter et al. [3] showed that patients who received a PECS II block had attenuated pain intensity at 6 and 24 h postoperatively. Although interpretative caution is required, this may be because the duration of local anesthesia is expected to be approximately 4–8 h. Blanco et al. [1] found that the analgesic duration of the PECS block lasted approximately 8 h postoperatively after the preoperative PECS block with ultrasound. Thomas et al. [9] reported that the average analgesic duration of the intraoperative PECS II block was 6 h after surgery. The results of our study were similar to those of previous reports, but the duration of the effect was shorter [2,3]. This difference may be because this study was conducted in patients who underwent robotic-assisted surgery, while previous studies were performed in open mastectomy.

Some drawbacks of preoperative ultrasound-guided PECS block are that it requires trained personnel to use ultrasound and that the procedure is time-consuming. In addition, Bakshi et al. [25] reported that a fluid-filled space along the fascial planes was observed when the preoperative PECS II block was applied. They also stated that the spread of the local anesthetic along the fascial plane may limit the use of electrocautery during surgical dissection. In the current study, the PECS II block was performed directly after RNSM without the use of ultrasound; thus, these drawbacks were resolved. The target structures of the PECS II block were dissected and easily exposed, allowing safer, faster, and more accurate targeting of the fascial plane.

There was no difference in the number of patients with PONV between the two groups, despite the significantly higher number of patients with a history of motion sickness in the PECS group than in the control group. In previous studies, no differences were observed in the incidence of PONV between the two groups despite a significant reduction in opioid requirements in the PECS group [2,3], which is consistent with results in the current study. However, in a previous study by Bashandy et al. [24], patients in the PECS group had significantly lower PONV scores than those in the control group. There are several risk factors for PONV, including female sex, history of motion sickness or PONV, smoking, and postoperative opioid use, which may have affected these results [26]. Thus, further prospective studies on PONV in the postoperative period are needed to draw definitive conclusions.

This study has several limitations, and the findings should be interpreted accordingly. First, this was a single-center trial with a relatively small sample size. However, RNSM is not frequently performed; thus, the results of the current study can be clinically meaningful, despite the small sample size. Second, opioid consumption and pain intensity were assessed only during the first 24 h after surgery, and there were no assessments of long-term outcomes such as chronic pain. Thus, further studies on the effect of the PECS II block on chronic pain are needed. Finally, in the current study, proper spread of the local anesthetic at the site of injection could not be confirmed visually when the block was performed. However, since the surgeon identified the exact anatomical structure and performed the injection, the local anesthetic agent infiltrated accurately in the appropriate target plane, leading to a significant reduction in the pain intensity of the PECS group. Nevertheless, based on the results of this study, better results may have been obtained if the actual infiltration process was visually confirmed through ultrasonography using sterilization cover. Although these limitations may be potential weaknesses of this study, to the best of our knowledge, this is the first study to examine the effect of an intraoperative PECS II block under direct vision through an open surgical incision in patients undergoing RNSM with IBR. It is clinically meaningful as it can provide effective postoperative analgesia in patients undergoing RNSM with IBR simply and safely through a surgical incision that has already been exposed during robotic mastectomy, without the familiarity of ultrasound or additional anesthesia procedures.

## 5. Conclusions

An intraoperative PECS II block under direct vision could provide effective analgesia and reduce postoperative 24-h opioid consumption in patients undergoing robotic NSM with prepectoral IBR using DTI or TE. Based on the results of this study, it would be worthwhile to proceed with multi-center, double-blinded, randomized clinical trials in the future.

## Figures and Tables

**Table 1:** Patient characteristics.

	Control Group (*n* = 15)	PECS Group (*n* = 15)	*p* Value
Age, years	43 ± 7	44 ± 7	0.734
BMI, kg/m^2^	22.3 ± 2.9	20.7 ± 3.3	0.164
ASA physical status			0.143
I	5 (33%)	9 (60%)	
II	10 (67%)	6 (40%)	
Smoking history			>0.999
Nonsmoker	14 (93%)	15 (100%)	
Exsmoker	1 (7%)	0 (0%)	
Menopause status			>0.999
Premenopausal	14 (93%)	13 (87%)	
Postmenopausal	1 (7%)	2 (13%)	
Motion sickness	0 (0%)	5(33%)	0.042 *
PONV history	0 (0%)	2 (13%)	0.483
Neoadjuvant chemotherapy	4 (27%)	1 (7%)	0.330

Data are presented as the mean ± standard deviation or number of patients (proportion). BMI, body mass index; ASA, American Society of Anesthesiologists; PONV, postoperative nausea and vomiting; PECS, pectoralis nerve II block. * *p* < 0.05.

**Table 2:** Intraoperative and surgical characteristics.

	Control Group (*n* = 15)	PECS Group (*n* = 15)	*p* Value
Anesthesia time, min	297 ± 47	295 ± 37	0.865
Operation time, min	252 ± 41	260 ± 41	0.573
Mastectomy time, min	146 ± 31	149 ± 33	0.765
Reconstruction time, min	103 ± 18	100 ± 31	0.756
Blood loss			>0.999
≤100 mL	13 (87%)	14 (93%)	
>100 mL	2 (13%)	1 (7%)	
Total fluid intake, mL	1943 ± 479	2155 ± 291	0.154
Urine output, mL	631 ± 597	646 ± 384	0.935
Intraoperative administered remifentanil, mg	0.8 ± 0.1	0.9 ± 0.3	0.417
Intraoperative administered phenylephrine, mg	4.1 ± 2.6	4.7 ± 1.3	0.475
Location			0.464
Right	9 (60%)	7 (47%)	
Left	6 (40%)	8 (53%)	
Type of reconstruction			0.682
Direct-to-implant	12 (80%)	10 (67%)	
Tissue expander insertion	3 (20%)	5 (33%)	
Lymph node procedure			>0.999
Sentinel lymph node biopsy	12 (80%)	11 (73%)	
Axillary lymph node dissection	3 (20%)	4 (27%)	
Specimen weight, g	349 ± 113	324 ± 141	0.612

Data are presented as the mean ± standard deviation or number of patients (proportion). PECS, pectoralis nerve II block.

**Table 3:** Postoperative characteristics.

	Control Group (*n* = 15)	PECS Group (*n* = 15)	*p* Value
Postoperative hospital stays, days	7.5 ± 1.8	6.7 ± 1.7	0.223
Patient satisfaction score	2.8 ± 0.4	3.5 ± 0.6	0.002 *
Nausea, n			0.486
None	11 (73%)	10 (26%)	
Mild	1 (7%)	3 (20%)	
Moderate	1 (7%)	2 (13%)	
Severe	2 (13%)	0 (0%)	
Vomiting, n	3 (20%)	3 (20%)	>0.999

Data are presented as the mean ± standard deviation or number of patients (proportion). PECS, pectoralis nerve II block. * *p* <0.05. Patient satisfaction score: 1 = very unsatisfied and 4 = very satisfied.

## Data Availability

The data presented in this study are available on request from the corresponding author.

## References

[1] Blanco R., Fajardo M., Parras Maldonado T. (2012). Ultrasound description of Pecs (modified Pecs I): A novel approach to breast surgery. Rev. Esp. Anestesiol. Reanim..

[2] Versyck B., van Geffen G.J., Chin K.J. (2019). Analgesic efficacy of the pecs II block: A systematic review and meta-analysis. Anaesthesia.

[3] Lovett-Carter D., Kendall M.C., McCormick Z.L., Suh E.I., Cohen A.D., De Oliveira G.S. (2019). Pectoral nerve blocks and postoperative pain outcomes after mastectomy: A meta-analysis of randomized controlled trials. Reg. Anesth. Pain Med..

[4] Neethu M., Pandey R.K., Sharma A., Darlong V., Punj J., Sinha R., Singh P.M., Hamshi N., Garg R., Chandralekha C. (2018). Pectoral nerve blocks to improve analgesia after breast cancer surgery: A prospective, randomized and controlled trial. J. Clin. Anesth..

[5] Blanco R. (2011). The ‘pecs block’: A novel technique for providing analgesia after breast surgery. Anaesthesia.

[6] Woodworth G.E., Ivie R.M.J., Nelson S.M., Walker C.M., Maniker R.B. (2017). Perioperative breast analgesia: A qualitative review of anatomy and regional techniques. Reg. Anesth. Pain Med..

[7] Toesca A., Peradze N., Galimberti V., Manconi A., Intra M., Gentilini O., Sances D., Negri D., Veronesi G., Rietjens M. (2017). Robotic nipple-sparing mastectomy and immediate breast reconstruction with implant: First report of surgical technique. Ann. Surg..

[8] Park H.S., Lee J., Lee D.W., Song S.Y., Lew D.H., Kim S.I., Cho Y.U. (2019). Robot-assisted nipple-sparing mastectomy with immediate breast reconstruction: An initial experience. Sci. Rep..

[9] Thomas M., Philip F.A., Mathew A.P., Jagathnath Krishna K.M. (2018). Intraoperative pectoral nerve block (pec) for breast cancer surgery: A randomized controlled trial. J. Anaesthesiol. Clin. Pharmacol..

[10] Hinchcliff K.M., Hylton J.R., Orbay H., Wong M.S. (2017). Intraoperative placement of pectoral nerve block catheters: Description of a novel technique and review of the literature. Ann. Plast. Surg.

[11] Haydon N.B., van der Rijt R., Downs C., Buckland G. (2016). A novel technique of intraoperative lateral pectoral nerve block during subpectoral breast implant placement. Plast. Reconstr. Surg. Glob. Open.

[12] Park H.S., Lee J., Lee H., Lee K., Song S.Y., Toesca A. (2020). Development of robotic mastectomy using a single-port surgical robot system. J. Breast Cancer.

[13] Lee J., Park H.S., Lee H., Lee K., Han D.H., Lee D.W. (2020). Axillary lymph node dissection using a robotic surgical system: Initial experience. J. Surg. Oncol..

[14] Sarfati B., Struk S., Leymarie N., Honart J.F., Alkhashnam H., de Fremicourt K.T., Conversano A., Rimareix F., Simon M., Michiels S. (2018). Robotic prophylactic nipple-sparing mastectomy with immediate prosthetic breast reconstruction: A prospective study. Ann. Surg. Oncol..

[15] Park H.S., Kim J.H., Lee D.W., Song S.Y., Park S., Kim S.I., Ryu D.H., Cho Y.U. (2018). Gasless robot-assisted nipple-sparing mastectomy: A case report. J. Breast Cancer.

[16] Williamson A., Hoggart B. (2005). Pain: A review of three commonly used pain rating scales. J. Clin. Nurs..

[17] Kaur U., Shamshery C., Agarwal A., Prakash N., Valiveru R.C., Mishra P. (2020). Evaluation of postoperative pain in patients undergoing modified radical mastectomy with pectoralis or serratus-intercostal fascial plane blocks. Korean J. Anesthesiol..

[18] Diab D.G., Elmaddawy A.A., Elganainy A. (2019). Intra-articular morphine versus dexmedetomedine for knee arthroscopy under local anesthesia. Anesth. Essays Res..

[19] Caljouw M.A., van Beuzekom M., Boer F. (2008). Patient’s satisfaction with perioperative care: Development, validation, and application of a questionnaire. Br. J. Anaesth..

[20] Kumar S., Goel D., Sharma S.K., Ahmad S., Dwivedi P., Deo N., Rani R. (2018). A randomised controlled study of the post-operative analgesic efficacy of ultrasound-guided pectoral nerve block in the first 24 h after modified radical mastectomy. Indian J. Anaesth..

[21] Kim D.H., Kim S., Kim C.S., Lee S., Lee I.G., Kim H.J., Lee J.H., Jeong S.M., Choi K.T. (2018). Efficacy of pectoral nerve block type ii for breast-conserving surgery and sentinel lymph node biopsy: A prospective randomized controlled study. Pain Res. Manag..

[22] Wang K., Zhang X., Zhang T., Yue H., Sun S., Zhao H., Zhou P. (2018). The efficacy of ultrasound-guided type II pectoral nerve blocks in perioperative pain management for immediate reconstruction after modified radical mastectomy: A prospective, randomized study. Clin. J. Pain.

[23] Versyck B., van Geffen G.J., Van Houwe P. (2017). Prospective double blind randomized placebo-controlled clinical trial of the pectoral nerves (pecs) block type II. J. Clin. Anesth..

[24] Bashandy G.M., Abbas D.N. (2015). Pectoral nerves i and ii blocks in multimodal analgesia for breast cancer surgery: A randomized clinical trial. Reg. Anesth. Pain Med..

[25] Bakshi S.G., Karan N., Parmar V. (2017). Pectoralis block for breast surgery: A surgical concern?. Indian J. Anaesth..

[26] Apfel C.C., Laara E., Koivuranta M., Greim C.A., Roewer N. (1999). A simplified risk score for predicting postoperative nausea and vomiting: Conclusions from cross-validations between two centers. Anesthesiology.

